# Improving oncology first-in-human and Window of opportunity informed consent forms through participant feedback

**DOI:** 10.1186/s12910-023-00890-4

**Published:** 2023-02-19

**Authors:** Anna M. Avinger, Hannah Claire Sibold, Gavin Campbell, Eli Abernethy, John Bourgeois, Tekiah McClary, Shannon Blee, Margie Dixon, R. Donald Harvey, Rebecca D. Pentz

**Affiliations:** 1 Wake Forest University School of Medicine, 475 Vine St, Winston-Salem, NC 27101 USA; 2 Duke University School of Medicine, 40 Duke Medicine Cir., Durham, NC 27710 USA; 3 Emory University Rollins School of Public Health, 1518 Clifton Rd, Atlanta, GA 30322 USA; 4 Winship Cancer Institute of Emory University, 1365 E Clifton Rd, Atlanta, GA 30322 USA; 5 South University Orlando Campus, 5900 Lake Ellenor Dr., Orlando, FL 32809 USA; 6 Creighton University Medical School, 2621 Burt Street, Omaha, NE 68178 USA; 7 Emory University School of Medicine, 201 Dowman Dr., Atlanta, GA 30322 USA; 8 Winship Cancer Institute, 2004 Ridgewood Dr., Office 301, Atlanta, GA 30322 USA

**Keywords:** Informed consent, Clinical trials, Window of opportunity, First-in-human

## Abstract

**Background:**

Although patient advocates have developed templates for standard consent forms, evaluating patient preferences for first in human (FIH) and window of opportunity (Window) trial consent forms is critical due to their unique risks. FIH trials are the initial use of a novel compound in study participants. In contrast, Window trials give an investigational agent over a fixed duration to treatment naïve patients in the time between diagnosis and standard of care (SOC) surgery. Our goal was to determine the patient-preferred presentation of important information in consent forms for these trials.

**Methods:**

The study consisted of two phases: (1) analyses of oncology FIH and Window consents; (2) interviews of trial participants. FIH consent forms were analyzed for the location(s) of information stating that the study drug has not been tested in humans (FIH information); Window consents were analyzed for the location(s) of information stating the trial may delay SOC surgery (delay information). Participants were asked about their preferred placement of the information in their own trial’s consent form. The location of information in the consent forms was compared to the participants’ suggestions for placement.

**Results:**

34 [17 FIH; 17 Window] of 42(81%) cancer patients approached participated. 25 consents [20 FIH; 5 Window] were analyzed. 19/20 FIH consent forms included FIH information, and 4/5 Window consent forms included delay information. 19/20(95%) FIH consent forms contained FIH information in the risks section 12/17(71%) patients preferred the same. Fourteen (82%) patients wanted FIH information in the purpose, but only 5(25%) consents mentioned it there. 9/17(53%) Window patients preferred delay information to be located early in the consent, before the “Risks” section.  3/5(60%) consents did this.

**Conclusions:**

Designing consents that reflect patient preferences more accurately is essential for ethical informed consent; however, a one-size fits all approach will not accurately capture patient preferences. We found that preferences differed for FIH and Window trial consents, though for both, patients preferred key risk information early in the consent. Next steps include determining if FIH and Window consent templates improve understanding.

**Supplementary Information:**

The online version contains supplementary material available at 10.1186/s12910-023-00890-4.

## Background

The informed consent process for clinical trials is essential to ensuring patient autonomy, understanding, and voluntariness [1]. While consent forms are meant to provide transparency and clarity, they often have low readability or are overly long with a significant amount of detail included [2]. Consent forms have become longer, more detailed, and more complex over time, and extensive length and complexity lead to lower patient understanding [3, 4]. When oncology consent forms were tested for readability at one cancer institute, it was found that none of the consent forms were written below an eighth-grade level [5], which is the recommended reading level for consent forms [6].

Currently, there are systems in place to assess and standardize the structure and process of informed consent, such as the institutional review board (IRB) and FDA guidelines. The National Cancer Institute (NCI) consent form template is used widely for cancer clinical trials and is the basis for some specific IRB templates. The NCI consent form template was created with the help of patient advocate groups, and the NCI consent form guidelines recommend using patient advocates to review consent forms [7]. Other research on the ethics of consent continues to push for the inclusion of patient input [8, 10], specifically, incorporating patients’ perspectives on consent form layout and presentation [11]. Since a template or guide for writing consent forms has been shown to improve patient understanding [12], the goal of this study was to create patient-preferred consent form templates for First in Human (FIH) and Window of Opportunity (Window) clinical trials. For clinical trials like these, with specific risks, a standard method of conveying those risks is important [13].

Risk-benefit analyses are often complex for FIH trials because there are no preliminary human safety data [14]. The level of risk to participants in these trials often relates to the degree of innovation of the biological pathway and/or delivery of the agent being investigated [15]. When novel drugs are used in tandem with approved agents, the FIH nature of the combination and safety risks are potentially more difficult and complicated to explain due to concerns such as overlapping adverse event profiles and drug interactions [16]. Regardless of the level of risk, all FIH trials are required to inform the patient of the known risks and communicate meaningful information about unknown risks [17]. While there have been several papers written on what should and should not be included in FIH consent forms [14, 17, 18] we found no reports of what patients would like to see in an informed consent for a FIH trial. Therefore, we aim to provide a FIH consent template that captures the patient perspective, with a focus on clarifying that patients, if they join the trial, will be on a first in human trial.

Likewise, Window trials have unique risks. In a Window trial, a treatment-naïve patient receives one or more investigational agents between the time they are diagnosed with cancer and their standard of care treatment (often surgery) [19]. The advantages of Window trials include expediting drug development [19, 20], improving understanding of pharmacodynamic parameters, and helping to identify biomarkers for patient selection for treatments. Window studies normally have endpoints of efficacy, pharmacodynamics, and safety [19]. However, the potential disadvantages include delaying surgery, safety and logistical barriers, and lack of patient benefit [19]. The risk of delaying standard of care surgery is a unique risk to Window trials; thus, it is critical that this information is clearly stated in the consent form [21]. Concerns about clear Window trial informed consent forms have been shared and discussed for decades [21]. More research is needed to develop tools to assess understanding of consent forms. The current project focuses on understanding the unique risk of Window trials—the risk delaying standard of care surgery. This project, therefore, assesses if Window consent forms include an explanation of the risk of delaying surgery and how patients would like this risk explained in the consent form.

## Methods

The study consisted of two phases: (1) analyses of consents for FIH and Window oncology trials open at a cancer center between 2019 and 2022; (2) interviews of patients who had reviewed consents for FIH or Window trials during their consent process.

### Ethics approval and consent to participate

The study was conducted under two protocols, one for the Window trials and one for the FIH trials. Both were approved by the Winship Cancer Institute Protocol Monitoring and Review Committee and by the Emory University Institution Review Board (Windows IRB99112187; FIH IRB00114961).

### Consent form analysis

Consents for FIH and Window oncology trials open to accrual at a cancer center from 2019 to 2022 were analyzed. Only dose escalation FIH trial consents, in which the investigational agent had never been tested in humans, were included. The consent forms for these trials were qualitatively analyzed for the location(s) of information stating that the study drug has not been tested in humans (FIH information) and the location of data from animal or other nonclinical testing. Window consents were analyzed for the location(s) of the definition of an investigational drug, the location(s) of the statement about potential benefits, and the location(s) of information stating the trial may delay SOC surgery (Delay information). Both types of consent were analyzed for the placement of the risks and trial schedule sections.

### Interview development

Qualitative interview questions were designed based on a review of the literature focusing on the unique risks of FIH and Window trials and how consent forms should present them. Both FIH and Window interviews were cognitively tested with cancer patients to assess clarity and completeness before finalization [22].

Participants were asked about their preferences for the placement and descriptions of the information in their own trial’s consent form. Eligibility required that the participants had reviewed their Window or FIH consent form, so multiple trial consent forms were reviewed. The participant was also provided with their own trial’s consent form during the interview, either as a paper copy or it was shown during video conferencing. Both interviews asked what information should be included in the consent form. Other key questions in each interview included:

#### FIH interview

1. What are the thoughts off the top of your head about first-in-human trials? If unclear, state that a first-in-human trial is one in which the drug being tested has never been used in humans before.
2. Where do you think the informed consent form should mention that the drug is being used for the first time in humans?
3. Is the information that researchers obtained from animal studies using the research drug in your trial important to you? If yes, would you want the side effects from the animal studies listed?

#### Window interview

1. Why did you choose to participate or not in this clinical trial? What were your concerns?
2. Was the timing of the trial clear to you?
3. Should the schedule of the trial go before or after the risks section?
4. Where do you think the risk of delaying standard of care surgery should go in the consent form?

### Recruitment

Patients offered enrollment in FIH or Window trials from October 2019 to February 2022 were eligible for the study. All of the FIH and Window trials limited enrollment to patients over 18 years old, so our cohort consisted only of adult patients. The disease-based working group research team approached each patient who had consented to a FIH or Window trial and asked for permission for the ethics team to offer them an interview study. The patient’s contact information was given to the ethics team with permission.

The ethics researcher then contacted the patient either by phone or in the clinic, explained the study in detail, shared the consent form, and obtained verbal consent. Once consented, the ethics researcher interviewed the participant via phone, video conference, or in-person at the clinic. As approved by the Emory University IRB, consent was documented by the completion of the interview. We planned to enroll patients until saturation of themes was reached for each of the types of trials, usually accomplished with 15–20 participants [23]. Given the limited number of FIH and Window trials, we did not sample for diversity but approached all patients who had consented to either a FIH or Window trial and who gave us permission to contact them.

### Qualitative analysis

Interviews were audio-recorded and qualitatively coded using standard multi-level semantic analysis [24]. After the first five interviews for each type of trial, two researchers identified major themes and types of answers independently (GC and RP for FIH, CS and RP for Windows). Code books for each trial type were finalized, and all the interviews were coded using the code books (GC, CS, AA). If a new code was discovered during the coding of subsequent interviews, the new code was added to the code book and all prior interviews were re-coded to determine if that code was applicable. All interviews were double coded (EA, SB, TM), and discrepancies were resolved by consensus. Descriptive statistics were used to summarize the demographic characteristics and responses to the interviews. The frequencies of the major themes mentioned were calculated [25].

## Results

We analyzed twenty FIH trial consents for six types of cancer (14 for any solid tumor, 2 for hematologic malignancies, 1 B-Cell lymphoma, 1 multiple myeloma, 1 gastrointestinal and 1 lung cancer), and we analyzed five Window trial consents for four types of cancer (1 lung, 1 gastrointestinal, 1 head and neck, and 2 breast cancer). Thirty-four (17 for each type of trial) of 42 (81%) patients approached participated. Four Windows patients and 2 FIH patients refused, and two other Windows patients were deemed ineligible because they had not reviewed the Window trial’s consent form. The 17 Window participants were on four different trials (5 on a lung cancer trial, 4 on a gastrointestinal trial, 7 on a head and neck trial and 1 on a breast cancer trial). The 17 FIH trial participants were on 10 different trials (13 on trials for solid tumors, 2 on a lung trial, 1 on a myeloma trial and 1 on a B-cell lymphoma trial). Saturation of themes was achieved with 17 participants.

Ten of the 17(59%) FIH trial participants understood they were on a FIH trial. Six of the 17(35%) FIH trial participants expressed concerns about the consent as a whole (3 wanted the consent to state more clearly that the drug was investigational, 4 found it confusing and disorganized, and one stated it was boring). Six of the 17(35%) Window trial participants expressed concerns about the consent as a whole (2 wanted a more detailed explanation of the study arms, 2 found the timeframe for the trial confusing, 5 found the language of the consent confusing and wanted it to use lay terminology and one stated that the consent should be more concise with less legalities added). Participants could mention more than one concern. When asked what should be included in an ideal consent, the standard consent elements were mentioned except that 4 of the 17 (24%) FIH participants wanted the specific benefit of this drug to be stated instead of the standard statement that the investigational agent may or may not benefit you. Three of the 17(18%) FIH participants wanted the time commitment of the trial stated in a simple sentence in addition to the multiple paragraphs describing the timeframe of procedures. And 4 of the 17 ( 24%) Window participants wanted the specific cost to them to be stated, which unfortunately would not be possible.

### First-in-human trial results

Patient preferences compared to the consent form placement for FIH trials are listed in Table 1. One consent form (5%) did not mention FIH information anywhere. Fifteen participants (88%) stated that they thought FIH information should be “one of the first things”/“up front”/first few pages. Eighteen (90%) of the consent forms listed the side-effects found in the animal studies conducted prior to the trials. Five (25%) mentioned animal/nonclinical data more than once. Our focus on FIH information is supported by the qualitative answers to the question of what should be included in the consent form with 8 of the 13(62%) comments identifying information about the drug, its risks and its benefits. Making it clear that the drug has never been used in humans before is key information about the drug and that risks and benefits are being extrapolated from animal studies.

**Table 1:** FIH info location in consent compared to patient preference

	Only in title	Only in purpose/introduction	Only in risks	Title & purpose	Title and risks	Purpose & risks	Title, purpose, & risks
Location of FIH Info in consent forms (n = 20)	0	0	20% (4)	0	5% (1)	20% (5)	10% (2)
Participant preference for location of FIH info (n = 17)	0	18% (3)	6% (1)	6% (1)	6% (1)	29% (5)	29% (5)

Fourteen (82%) FIH participants thought it is important to include preliminary data from animal/nonclinical trials in consent forms, and 10 (58%) participants wanted to know the side effects identified during animal/nonclinical trials. Twelve of 17 (71%) of participants thought that side effects found in animal studies should go in the risks section rather than earlier in the document. Two (10%) of the 20 FIH consent forms mentioned the animal data in the Purpose, 7 (35%) in the section on study drug assignment and dosing, 9(45%) in the risks section and 2(10%) did not mention animal data.

To summarize, participants preferred FIH information to be included in the purpose of the consent form and in the risks section. Data from animal/nonclinical trials should be included in the consent form, but the specific location of this information can vary. Possible side effects of the study drug should be included in the risks section.

Based on the patient interviews, we propose an FIH consent form template (see Additional file 1) including patient-preferred locations for FIH information and animal/nonclinical data. We used Winship Cancer Center’s IRB-required consent form template as the basis for our template [26].

### Window trial results

Patient preferences compared to the consent form placement for Window trials are listed in Table 2. Five Window trial informed consent forms were in use during 2019–2022 at the Winship Cancer Center. All five consent forms included the information that there is no guarantee the patient will benefit from the study in the benefits section.

**Table 2:** Delay information for Window trials location in consent compared to patient preference

	Key concepts	Purpose	Trial schedule	Beginning of risks section	End of risks section
Location of delay information in consent forms (n = 5)	20% (1)	40% (2)	0	0	40% (2)
Participant preferences for location of delay information (n = 17)	18% (3)	29% (5)	6% (1)	18% (3)	24% (4)

One Windows participant had no preference

Patient preference for the location of the risk of delaying surgery (delay information) varied (Table 2). However, it should be noted that while 41% (7/17) of participants thought delay information should be located in the risk section, 53% (9/17) wanted the information stated earlier in the consent form. For those participants on the trials whose consent had delay information at the end of the risks section, 7/11 (64%) wanted the information to be stated earlier. Our focus on delay information is supported by the qualitative answers to the question of what should be included in the consent form with 9 of the 28(32%) comments identifying risks of the trial, and 3 (11%) comments specifically stating the potential to delay surgery should be stated explicitly in the consent. Another 6 (21%) of the comments mentioned that the timeframe of the trial should be included.

Participants made additional suggestions for the placement of different items in the consent and about format. Fourteen of 17(82%) thought the term “investigational drug” should be explained in the introduction or purpose of the consent form. Fourteen (82%) agreed with the NCI consent form template in that the study schedule should come before the risks section. Twelve (71%) of the participants wanted the trial schedule to be presented in an outline/bullet-point format. When possible, the informed consent form should include a one-page summary, or key concepts page of the unique risks and schedule timeline, as 11 (79%) of the participants said this would be helpful. Three of the 5(60%) Window consent explained ‘investigational drug” in the purpose. All five consents followed the NCI template, placing the trial schedule before the risks. Four of the five (80%) consents bulleted the trial schedule as the patients preferred and only two (40%) of the five had a key concepts page.

Based on the patient interviews, we propose a Window trial consent form template (see Additional file 2) including patient-preferred locations for delay information and an explanation of an investigational drug. We used Winship Cancer Institute’s IRB-required consent form template as the basis for our template [26].

## Discussion

Location of information in consent forms is important, as it has been shown that patients tend to read certain sections of consent forms more attentively than others [27, 28], though the impact of the location of information on the comprehensibility of key ideas in consent forms needs to be studied further [27]. Douglas et al. [28] demonstrated that the first two sections of a consent form are the most thoroughly read. This finding, if correct, shows the importance of our results, as most participants wanted important information to be closer to the beginning of the consent form. FIH patients wanted FIH information in the purpose, and Windows patients generally wanted delay information to be earlier on in the consent form than at the end of the risks section. Understanding where patients prefer information is important in making sure consent forms are patient-centric, and for FIH and Window trials, the risks are complex and thus need special attention. Creating patient-preferred consent form templates is an important step in ensuring clarity and understanding for patients considering clinical trials [11, 12], particularly for complex trials, such as FIH and Window trials.

For FIH trials, over half of the participants (59%) thought that FIH information should be included more than once. This is important because it is known that repeating information can lead to better understanding and memory [29, 30]. In specific studies on the consent process, repetition of information through different modes of presentation improved understanding and retention [31, 32]. Therefore, it could be beneficial to include important information, such as unique risks and side effects more than once and in different ways. Thus, although there was not a consensus among Window participants to include delay information more than once in the consent forms, consideration should be made to do so, as the patient-preference for exact location varied, and it may lead to better understanding and memory.

This study also addressed the acceptability of the NCI informed consent form template. For complex trials, such as FIH and Window trials, the NCI template is generally acceptable, as 82% (14/17) agreed with the NCI template in that the study schedule should come before the risks section and 79% (11/14) of the participants said that a one-page summary or key concepts page would be helpful. However, consent form authors should consider including explanations of complex information at the beginning of the consent forms to help increase patient understanding. Our next research study will be to test our templates with FIH and Window clinical trial patients to see if the templates improve understanding and retention of key information in consent forms. If effective, we will provide our templates to our IRB and widely distribute through publication.

Given the low accrual goals of Window and FIH trials and the limited number of Window trials conducted, the sample size for this study is small. This limitation was particularly concerning for the Window consent form analysis, as there were only 5 Window consents. However, we did reach saturation of themes for the interview data, which is the standard in qualitative analyses. Since our aim was to focus on the unique risks of these two types of trials, we did not do a thorough assessment of patient understanding of and preferences for adequate consents, which are good topics for future research. In addition, as a single institution study, the results may not be generalizable. Finally, we have not yet tested the templates, so their acceptability is not yet established.

## Conclusions

Based on participant preference, key information about FIH and Window trials should be stated early in the consent forms. A statement that the investigational agent has never been used in humans should be included at the beginning of FIH consent forms. For Window trials, the risk of delaying surgery should be included in the purpose or key concepts page of Window trial consent forms as well as in the risks section. The study schedule should be placed before the risks section in alignment with the NCI consent form template. It is valuable to include key information in several sections of the consent. Incorporating patient preferences when creating new templates is critical to ensure the consent form is clear, informative, and remains patient-centered.

## Supplementary Information

**Additional file 1**. Template 1. FIH trial consent form template.

**Additional file 2**. Template 2. Window trial consent form template.

## Data Availability

The datasets generated during and analyzed during the current study are not publicly available because publicizing interview transcripts may breach participant confidentiality, but de-identified data sets are available from the corresponding author on reasonable request.

## Abbreviations

FIH: First in human
SOC: Standard of care
NCI: National Cancer Institute
FDA: Food and Drug Administration
IRB: Institutional Review Board
FIH information: Information stating that the study drug has not been tested in humans
Delay information: Information stating the window trial may delay standard of care (SOC) surgery
