# Supplementary material for: Improving oncology first-in-human and Window of opportunity informed consent forms through participant feedback
Source: BMC Med Ethics. 2023 Feb 19;24:12. doi: 10.1186/s12910-023-00890-4 (PMC9938963; doi:10.1186/s12910-023-00890-4)
Supplement: Supplementary file 1 — Additional file 1. Template 1. FIH trial consent form template. [file 12910_2023_890_MOESM1_ESM.pdf]

Template 1. FIH Trial Consent Form Template

**First-in-Human (optional)** Research Title		
Key concepts		
	Why is this study being done?	
		**FIH information**
	Do you have to be in the study?	
	What do I have to do if I choose to participate in this study?	
	How is this study going to help you?	
	What are the risks or discomforts I should know about before making a decision?	
		**FIH information**
	Alternatives to joining this study	
	Costs	
	What should I do next?	
Title		
IRB #		
Principle Investigator		
Sponsor		
Investigator-sponsor		
Study-supporter		
Introduction		
What is the purpose of this study?		
	**FIH information**	
What will I be asked to do?		
How will my medicine be provided?		
Who owns my study information and samples?		
What are the possible risks and discomforts?		
	**FIH information**	
	**Data from animal/nonclinical trials**	
	**Side effects**	
Will I benefit directly from the study?		
Will I be compensated for my time and effort?		
What are my other options?		
How will you protect my private information that you collect in this study?		
Certificate of Confidentiality		
Storing and sharing your information		
Medical record		

In case of injury
Costs
Withdrawal from the study
Authorization to Use and Disclose Protected Health Information
Consent and Authorization
